# Perioperative Anaphylaxis and Respiratory Failure During AVF Surgery in an End‐Stage Renal Disease Patient

**DOI:** 10.1002/ccr3.73809

**Published:** 2026-11-02

**Authors:** Madeline Dow, Aariya Srinivasan, Sabry Ayad

**Affiliations:** ^1^ Department of Anesthesiology Cleveland Clinic Foundation Cleveland Ohio USA; ^2^ Department of Anesthesiology Outcomes Research Consortium Cleveland Ohio USA; ^3^ Cleveland Clinic, Fairview Hospital Cleveland Ohio USA

**Keywords:** allergy, anesthesia, critical care medicine, nephrology

## Abstract

Perioperative anaphylaxis can cause sudden, catastrophic respiratory and cardiovascular collapse in high‐risk ESRD patients, even during regional anesthesia. Early recognition, prompt epinephrine administration, serum tryptase measurement, vigilant respiratory monitoring, and multidisciplinary preparedness—including ECMO capability—are critical to improving survival and preventing recurrent perioperative events.

## 1 Introduction

Respiratory failure during anesthesia is a rare but potentially life‐threatening complication. It occurs in up to 5%–10% of high‐risk patients and accounts for significant perioperative morbidity and mortality, particularly in patients with preexisting conditions [1]. Patients with end‐stage renal disease (ESRD) are at heightened risk due to fluid overload, uremic pulmonary dysfunction, anemia, and metabolic imbalances [2]. In addition to these physiologic vulnerabilities, ESRD patients remain susceptible to perioperative anaphylaxis, a rapid IgE‐mediated reaction in which mast cells and basophils degranulate, releasing mediators such as histamine, tryptase, and platelet‐activating factor. The resulting bronchospasm, vasodilation, and capillary leak can swiftly progress to cardiovascular collapse [3, 4, 5]. In ESRD patients undergoing dialysis access creation, regional anesthesia is frequently used for AVF surgery because it offers superior vascular outcomes and primary patency rates compared to local anesthesia [2]. We present the case of a 25‐year‐old ESRD patient undergoing AVF revision surgery who experienced sudden cardiac arrest. She had experienced a prior perioperative cardiac arrest in which rocuronium‐associated bronchospasm was suspected, although the causative agent was not definitively established.

## 2 Case History and Examination

A 25‐year‐old female with ESRD on thrice‐weekly hemodialysis via a right internal jugular (IJ) tunneled dialysis catheter was scheduled for revision of her right upper‐extremity arteriovenous fistula. Her past medical history included type 1 diabetes mellitus, hypertension, asthma, deep venous thrombosis, and peptic ulcer disease. Approximately 10 months before the current hospitalization, she experienced a cardiac arrest during anesthetic induction for dialysis access surgery. Shortly after induction, she developed severe bronchospasm with difficulty in ventilation, progressing to respiratory arrest and pulseless electrical activity. She required cardiopulmonary resuscitation with multiple rounds of chest compressions, three doses of epinephrine, and endotracheal intubation, after which she stabilized and was transferred to the surgical intensive care unit. The treating team documented suspected rocuronium‐associated histamine release with severe bronchospasm as a potential precipitating mechanism, although the etiology was not definitively established. She also had a history of postoperative nausea and vomiting, as well as difficult intravenous (IV) access.

## 3 Investigations and Diagnosis

In preoperative holding, the patient received right brachial plexus and intercostobrachial single‐shot nerve blocks for regional anesthesia and perioperative analgesia. The brachial plexus block included 6.5 mL of 0.5% bupivacaine, 2 mg dexamethasone, and 50 mg of 1% lidocaine; the intercostobrachial block included 3.5 mL of 0.5% bupivacaine, 2 mg dexamethasone, and 50 mg of 1% lidocaine. Bupivacaine and lidocaine served as the local anesthetic components, while dexamethasone was administered as an adjunct to prolong block analgesia.

In the operating room, she was placed supine and received a single 1‐mg dose of midazolam for sedation while the right arm was prepared. Her vitals were as follows: blood pressure 130/82 mmHg, pulse 81 beats/min, and SpO_2_ 89% with a glucose level of 111 mg/dL. Approximately 40 min after administration of the regional anesthetic medications and approximately 10 min after administration of intravenous sedation, she reported feeling hot and nauseated. Measured glucose levels were 117 mg/dL. She then became hypoxic and minimally responsive. Her blood pressure fell to 88/43 mmHg, with a pulse of 82 beats/min and SpO_2_ of 54%. Despite 2‐hand bag‐mask ventilation, she progressed to pulseless electrical activity arrest.

## 4 Management

Advanced cardiac life support was initiated immediately with chest compressions, vasopressors, and endotracheal intubation using a 7.0 mm cuffed tube. Endotracheal intubation proved difficult due to copious secretions; three GlideScope attempts resulted in a right soft‐palate laceration. Right femoral arterial and venous lines were placed during resuscitation, and intermittent return of spontaneous circulation (ROSC) was achieved on epinephrine and norepinephrine infusions. Despite sustained ROSC, she remained severely hypoxic (PaO_2_ < 40 mmHg) and hypercapnic (PaCO_2_ > 100 mmHg) with refractory metabolic acidosis (serum pH 6.9). Serum tryptase levels were 137 μg/L (reference range < 8.4 μg/L). Epinephrine was administered as repeated boluses during cardiopulmonary resuscitation and subsequently as a titrated infusion for persistent hemodynamic instability, with norepinephrine added for additional vasopressor support. In total, she received 5144 μg of epinephrine via 0.1 mg/mL bolus injections, 1785 μg of epinephrine from a 4 mg/250 mL 0.9% NaCl infusion, and 737 μg of norepinephrine from a 16 mg/250 mL infusion. Veno‐venous extracorporeal membrane oxygenation (VV ECMO) was initiated via bilateral femoral cannulation with a flow of 2.82 L/min, a pump speed of 2525 revolutions/min, and a sweep gas of 4 L/min. VV ECMO settings were maintained with FiO_2_ 60%, SpO_2_ 88%–92%, and PaO_2_ 75 mmHg.

## 5 Outcome and Follow‐Up

Over a 17‐day hospitalization, with the cardiac arrest designated as day 0, she underwent multiple bronchoscopies revealing submucosal erythema, mucus plugging, and thick cloudy secretions. Bronchoalveolar lavage cultures were negative. The right soft palate laceration was sutured, bleeding was managed with a 500 mg dose of 5% tranexamic acid oral rinse, and a course of amoxicillin‐clavulanate was administered. VV ECMO was successfully decannulated on day 3. On day 4, she was extubated to nasal cannula, with a documented arterial blood gas showing a pH of 7.38, PaCO_2_ of 52 mmHg, PaO_2_ of 109 mmHg, and bicarbonate of 30 mmol/L. She subsequently developed worsening hypoxemia, requiring escalation to high‐flow nasal cannula on day 6. Noninvasive ventilation was not tolerated because of agitation. On day 7, worsening respiratory failure with tachypnea and a PaO_2_ of 48 mmHg necessitated re‐intubation. Empiric piperacillin‐tazobactam and vancomycin were started pending culture results. Transthoracic echocardiography showed preserved biventricular function with a left ventricular ejection fraction of 60% and a normal right ventricle. She was successfully extubated to nasal cannula on day 11 and remained stable. Hemodialysis was continued via her existing right IJ tunneled catheter thrice weekly. The patient was referred for outpatient allergy evaluation and skin testing; however, confirmatory allergy testing was not completed. Consequently, the specific causative perioperative agent could not be definitively identified.

## 6 Discussion

Multiple factors may have contributed to the patient's second episode of respiratory failure, including phrenic nerve involvement, oversedation, airway obstruction, and anaphylaxis. Phrenic nerve blockage causing hemidiaphragmatic paralysis is a complication associated with supraclavicular brachial plexus blocks. Even with ultrasound guidance, standard volumes may produce hemidiaphragmatic paralysis in 34%–70% of patients [6]. In healthy individuals, it is usually clinically silent, but in high‐risk populations such as anemic, fluid‐overloaded ESRD patients, the decrease in ventilation can lead to significant respiratory compromise.

Oversedation from anesthetics can further compound the risk of respiratory failure. Anesthetics such as midazolam act centrally to depress hypoxic and hypercapnic ventilatory drives, which can lead to significant respiratory depression, exacerbating the risks inherent in regional anesthesia performed in settings with potentially suboptimal monitoring [7]. This patient's preexisting asthma and renal impairment increased the risks of respiratory depression with standard anesthetic doses. Adverse respiratory events related to oversedation are common in nonoperating room anesthesia, such as preoperative holding, where a lack of continuous monitoring may result in compromised ventilation that goes unrecognized until severe complications arise [7]. Accordingly, clinicians should ensure that complex patients, such as those with ESRD, receive appropriate respiratory monitoring to detect and correct any ventilatory compromise without delay.

Airway obstruction is another critical factor to consider. Upper airway obstruction often occurs due to reduced airway muscle tone from anesthetics and opioids, leading to hypoxemia and hypercapnia [8]. Mechanical obstruction secondary to AVF manipulation, such as thrombus formation or embolism, can further compromise circulation and ventilation. In one case report, manual thrombus mobilization during AVF manipulation led to pulmonary embolism and cardiac arrest, underscoring the complexity of potential vascular complications during these procedures [9]. In our patient, the planned fistula revision had not been performed before the arrest, and there was no imaging evidence of pulmonary embolism, making this mechanism less likely.

Finally, perioperative anaphylaxis must also be considered given the abrupt onset of symptoms, profound hypoxemia and hypotension, cardiovascular collapse, and markedly elevated serum tryptase [3, 4, 5]. The allergy and immunology consultation recommended temporary avoidance of the perioperative agents involved in the current and prior reactions pending formal outpatient testing, without identifying a specific causative agent. Rocuronium had been considered a possible contributor to the patient's prior perioperative arrest because of suspected severe bronchospasm following its administration. However, this association was never confirmed by allergy testing, and rocuronium was not administered during the current event of cardiac arrest. Therefore, the causative agent in the current reaction remains uncertain. Formal post‐event allergy evaluation is important to identify a potential culprit and guide future anesthetic management.

Management strategies in these scenarios include immediate recognition, supportive interventions such as advanced cardiac life support, and the potential implementation of extracorporeal membrane oxygenation (ECMO) in refractory cases. Selecting anesthetics with favorable cardiovascular safety profiles, such as ropivacaine over bupivacaine, can reduce the risk of severe cardiotoxicity in regional anesthesia [10]. Anesthetic planning should favor techniques that minimize phrenic spread, such as infraclavicular rather than supraclavicular approaches, and employ the lowest effective local anesthetic volumes [6]. Given the complexity of anesthesia in ESRD patients, comprehensive preoperative evaluation using risk stratification tools is recommended to identify those at high risk for respiratory and cardiac complications [1]. Optimizing perioperative management through multidisciplinary collaboration and patient‐specific anesthetic planning can significantly mitigate risks and improve patient outcomes.

Several limitations should be considered when interpreting this case. Much of the clinical course, including details of the patient's prior perioperative arrest, was reconstructed retrospectively from medical documentation, which limits the ability to fully characterize all aspects of the events and clinical decision‐making. Although the abrupt clinical deterioration and markedly elevated serum tryptase supported the diagnosis of perioperative anaphylaxis, confirmatory post‐event allergy testing was not completed. Because multiple perioperative exposures occurred in the setting of the current reaction, the specific causative agent could not be definitively identified. Accordingly, attribution of either event to a specific perioperative agent should be interpreted with caution.

## 7 Conclusion

This case underscores the multifactorial challenges of perioperative anesthetic management in patients with ESRD, in whom severe respiratory and cardiovascular deterioration may have multiple contributing factors. In this patient, the abrupt onset of profound hypoxemia, hypotension, cardiovascular collapse, and markedly elevated serum tryptase supported perioperative anaphylaxis, although the specific causative agent could not be identified. Early recognition, prompt advanced life support, and VV ECMO in the setting of refractory hypoxemia were critical to stabilization. Careful preoperative assessment, review of prior anesthetic exposures, formal post‐event allergy evaluation, and individualized multidisciplinary planning are important to guide future anesthetic care and reduce the risk of recurrent perioperative reactions.

## Author Contributions

**Madeline Dow:** conceptualization, writing – original draft, writing – review and editing. **Aariya Srinivasan:** conceptualization, writing – original draft, writing – review and editing. **Sabry Ayad:** conceptualization, supervision, writing – review and editing.

## Funding

The authors have nothing to report.

## Consent

Written informed consent was acquired from the patient whose case details are written in the study to publish this report in accordance with the journal's patient consent policy.

## Conflicts of Interest

The authors declare no conflicts of interest.

## Data Availability

The data supporting this report's findings are available on request from the corresponding author. The data are not publicly available due to privacy or ethical restrictions.

## References

[1] J. Palermo , S. Tingey , A. K. Khanna , and S. Segal , “Evaluation and Prevention of Perioperative Respiratory Failure,” Journal of Clinical Medicine 13, no. 17 (2024): 5083, 10.3390/jcm13175083. PMC11396761 39274295

[2] C. Gao , C. Weng , C. He , J. Xu , and L. Yu , “Comparison of Regional and Local Anesthesia for Arteriovenous Fistula Creation in End‐Stage Renal Disease: A Systematic Review and Meta‐Analysis,” BMC Anesthesiology 20, no. 1 (2020): 219, 10.1186/s12871-020-01136-1. PMC7457346 32867692

[3] P. Dewachter , C. Mouton‐Faivre , and C. W. Emala , “Anaphylaxis and Anesthesia: Controversies and New Insights,” Anesthesiology 111, no. 5 (2009): 1141–1150, 10.1097/ALN.0b013e3181bbd443. 19858877

[4] A. Dodd , P. J. Turner , J. Soar , L. Savic , and UK Perioperative Allergy Network , “Emergency Treatment of Peri‐Operative Anaphylaxis: Resuscitation Council UK Algorithm for Anaesthetists,” Anaesthesia 79, no. 5 (2024): 535–541. 10.1111/anae.16206 38205901

[5] T. Takazawa , K. Yamaura , T. Hara , T. Yorozu , H. Mitsuhata , and H. Morimatsu , “Practical Guidelines for the Response to Perioperative Anaphylaxis,” Journal of Anesthesia 35, no. 5 (2021): 778–793, 10.1007/s00540-021-03005-8. 34651257

[6] J. Lang , X. Cui , J. Zhang , and Y. Huang , “Dyspnea Induced by Hemidiaphragmatic Paralysis After Ultrasound‐Guided Supraclavicular Brachial Plexus Block in a Morbidly Obese Patient,” Medicine (Baltimore) 101, no. 2 (2022): e28525, 10.1097/MD.0000000000028525. PMC8758049 35029208

[7] I. E. Royz , N. B. Clevenger , A. Bochenek , A. R. Locke , and S. B. Greenberg , “Respiratory Depression in Non‐Operating Room Anesthesia: An Overview,” Journal of Clinical Medicine 14, no. 13 (2025): 4528, 10.3390/jcm14134528. PMC12250410 40648902

[8] M. Karcz and P. J. Papadakos , “Respiratory Complications in the Postanesthesia Care Unit: A Review of Pathophysiological Mechanisms,” Canadian Journal of Respiratory Therapy 49, no. 4 (2013): 21–29. PMC4456822 26078599

[9] M. Gandolfi and B. S. Fernando , “Cardiac Arrest Following Arteriovenous Fistula Manipulation—A Cautionary Note,” BMJ Case Reports 2017 (2017): bcr‐2017‐220179, 10.1136/bcr-2017-220179. PMC5534971 28611138

[10] D. Viderman , B. Ben‐David , and A. Sarria‐Santamera , “Analysis of Bupivacaine and Ropivacaine‐Related Cardiac Arrests in Regional Anesthesia: A Systematic Review of Case Reports,” Revista Española de Anestesiología y Reanimación 68, no. 8 (2021): 472–483, 10.1016/j.redare.2020.10.005. 34538765

